# Burnout and Associated Risk Factors a Cross-Sectional Study Among Staff of Radiology Clinics in Slovakia

**DOI:** 10.3390/healthcare14152385

**Published:** 2026-08-04

**Authors:** Michal Slašťan, Anna Lesňáková, Peter Kubiš

**Affiliations:** 1 Department of Health Disciplines, St. Elizabeth University of Health and Social Work, 810 06 Bratislava, Slovakia; kubisp@uvn.sk; 2 Faculty of Health, Catholic University, 034 01 Ružomberok, Slovakia; anna.lesnakova@ku.sk; 3 SNP Central Military Hospital, Faculty Hospital, 034 01 Ružomberok, Slovakia

**Keywords:** occupational burnout, radiologists, radiographers, Burnout Assessment Tool, workload, shift work, Slovakia

## Abstract

**Background:** With the rising demand for fast and reliable diagnosis the radiology departments struggle to keep up. In Slovakia however it is difficult to find more employees for radiology clinics and departments, therefore the employees are ever more overburdened, which may lead to burnout. **Objective:** The aim of our study was to assess the burnout situation among radiology clinics and departments employees and asses the specific causes that increase the risk of burnout. Compare the burnout profile of radiologists and technicians. And look for potential targets for intervention in order to stabilize and improve the situation. **Methods:** Standardized Burnout assessment tool (BAT), validated in Slovak and an author-developed exploratory instrument were distributed among employees of ten hospitals in Slovakia. Convenience sampling was used in ten radiology clinics and departments in big, small and specialized hospitals. The entire questionnaire consisted of 55 questions. The received responses were processed statistically and correlations were analyzed. **Results:** There have been 213 questionnaires distributed and 105 complete and valid responses received. 26 respondents (24.76%) had an overall BAT score at or above 3.02 and can be considered at risk of burnout. The dominants dimensions of burnout in our set were exhaustion (3.06) and cognitive impairment (2.76). The highest score in the author-developed exploratory instrument was in visual exhaustion of radiologists (3.78). In the group of workers with 5 or more 24 shifts monthly mental distance is elevated (3.05). Burnout profiles in equal groups (*N* = 46) of radiologists and radiology technicians were compared with significantly higher levels of cognitive impairment found in radiologist. **Conclusions:** In our study 24.76% of respondents can be considered in risk of burnout, with highest scores in exhaustion and cognitive impairment dimensions, emotional impairment was relatively lower. Cognitive impairment seems to be more prevalent among radiologist compared to radiology technicians. Higher number of 24-h shifts was associated with higher mental distance scores. The highest Likert scale scores in the author developed exploratory instrument are in visual exhaustion, professional isolation and the feeling of anonymous service.

## 1. Introduction

Over the past two decades, radiologists have experienced a substantial increase in workload driven by the growing utilization of advanced imaging modalities and the increasing complexity of image interpretation. Although the number of imaging examinations has risen steadily, the number of images requiring interpretation has increased even more rapidly, particularly for CT and MRI, substantially increasing cognitive workload per radiologist [1]. Recent studies have shown that image volume per radiologist increased nearly fourfold between 2009 and 2022, while professional workload measured by relative value units also increased significantly despite relatively stable examination numbers [2]. These trends have been identified as important contributors to occupational stress, diagnostic fatigue, and burnout among radiology professionals. Furthermore, typical examinations are getting more detailed with more scans of increasing detail and in case of MRI examinations more scan series as well. This additional workload including the 24 h shifts compounded by the changes to environment and the ergonomics of the workplace creates a situation where the employees of radiology departments are at risk of burnout [3,4,5].

Burnout is defined by World Health Organization (WHO) as an occupational phenomenon resulting from chronic workplace stress that has not been successfully managed. Since the 1970s and 1980s it has been investigated across a wide range of professions, with healthcare workers consistently identified as one of the highest-risk groups [6]. The COVID-19 pandemic further intensified attention to burnout among healthcare professionals. Increased workload, frequent exposure to suffering and death, ethical dilemmas, high emotional involvement and pressure for greater productivity created conditions in which emotional exhaustion and burnout became increasingly prevalent [1,7,8]. In many healthcare institutions in Slovakia, these factors were compounded by understaffing, excessive administrative demands, prolonged work shifts, and inadequate financial compensation [9,10].

The doctors in the radiology department are responsible for aiding patient diagnosis by analyzing thousands of scans daily and distinguishing pathologic changes in the morphology. The analysis requires focus on the console monitor and concentration. A doctor suffering from burnout has impaired focus and lacks the necessary concentration, which may lead to increased occurrence of mistakes and may lead to poor outcomes for the patients [11]. In one meta-analysis, up to 82.9% of radiologists/trainees had at least one positive MBI subscale, 55.5% had two, and 16.7% had three. A systematic review reports overall burnout prevalence in radiology of 33–88% [12,13]. Studies on radiographers and specialized radiologists (interventional, cardiothoracic) show high levels of exhaustion, depersonalization, low sense of accomplishment, and high stress [1,14]. Key determinants include workload, overtime, night shifts, staff shortages, technological burden and isolation, productivity pressure, and poor work–life balance. Addressing burnout requires both individual- and organization-level interventions to ensure sustainable healthcare delivery [15,16]. In the setting of Slovak healthcare shortage of staff is a serious problem for many hospitals, clinics and departments, it is reported to be more serious for nurses, but also concerns other occupations [10]. The studies of what impact this has on the employees working in the system and whether any interventions would benefit the staff of healthcare institutions are however not regularly done.

The aim of this study was to assess burnout among employees of radiology departments in Slovakia using the Burnout Assessment Tool (BAT) and to identify radiology-specific occupational factors associated with burnout through an author-developed exploratory questionnaire. A secondary aim was to evaluate the relationships between burnout dimensions and selected demographic and occupational characteristics. It is the first study done in the setting of radiology clinics and departments in Slovakia using the BAT methodology.

## 2. Materials and Methods

### 2.1. Study Design

This study employed a cross-sectional, questionnaire-based design to assess the prevalence and determinants of burnout among employees working in radiology departments and workplaces in Slovakia. Data collection was conducted using a standardized psychometric instrument (Burnout Assessment Tool; BAT (developed by Wilmar B. Schaufeli and colleagues, KU Leuven, Leuven, Belgium.)) combined with an author-developed exploratory questionnaire targeting radiology-specific occupational stressors. The study was conducted during the January and February of 2026. The following hospitals and institutes were selected for our survey:

Žilina region

- Central military hospital SNP Ružomberok—Faculty Hospital,
- Lower Orava Hospital and Polyclinic MUDr. L. N. Jégého Dolný Kubín,
- Upper Orava Hospital and Polyclinic Trstená,
- Liptov Hospital with Polyclinic MUDr. Ivana Stodolu Liptovský Mikuláš.

Prešov region

- University Hospital with Polyclinic J. A. Reimana Prešov.

Košice region

- University Hospital Louis Pasteura Košice,
- Eastern Slovak Institute of Cardiovascular Diseases (VÚSCH) Košice, a. s.

Bratislava region

- Bory Penta Hospitals, Bratislava—Bory. Banská Bystrica region
- Faculty Hospital and Polyclinic F. D. Roosevelta Banská Bystrica,
- Central Slovak Institute of Cardiovascular Diseases (SÚSCCH) Banská Bystrica, a. s.

### 2.2. Study Instruments

#### 2.2.1. General Questions

The initial section of the questionnaire consisted of six general items assessing participant characteristics and study eligibility. Participants were asked to confirm consent to participate in the research, followed by demographic and professional questions including sex, age, years of experience in healthcare, professional position, and highest level of completed education. These variables were used to describe the study sample and evaluate potential differences in burnout characteristics between occupational and demographic groups.

#### 2.2.2. Burnout Assessment Tool (BAT)

The Burnout Assessment Tool conceptualizes burnout as a work-related syndrome comprising four core dimensions: **exhaustion**, **mental distance**, **cognitive impairment**, and **emotional impairment**, as well as secondary symptoms including **psychological distress** and **psychosomatic complaints** [17]. Previous studies have demonstrated good psychometric properties, including construct validity, internal consistency, and measurement invariance across demographic and cross-national samples [18]. In our study we used Full BAT-33 version which consists out of BAT-C (Core symptoms) with 23 items and BAT-S (Secondary symptoms) with 10 items. We employed a Slovak language version, which was culturally adapted and validated in a prior study [19].

#### 2.2.3. Author-Developed Exploratory Instrument

To capture radiology-specific occupational exposures, an exploratory questionnaire was developed by the authors. To specify the relevant factors three heads of radiology departments were consulted. The author-developed exploratory instrument consisted of 16 items structured into four domains:

1. **Technostress and cognitive workload** (3 items)
  - I experience anxiety or tension when new versions of diagnostic software (PACS/RIS) and new imaging equipment are introduced.
  - I perceive the number of artifacts and technical errors during examinations as a frustrating factor that slows down my work.
  - I feel that expectations regarding my work speed are increasing faster than the performance of the technologies I use.

All of these are answered by a frequency Likert scale.

2. **Physical and ergonomic factors** (7 items)
  - Working in an environment with insufficient natural daylight negatively affects my mood and energy levels.
  - During my work shift, I experience significant visual fatigue (eye strain, burning eyes, blurred vision) as a result of prolonged monitor use.
  - Noise from imaging equipment (especially MRI scanners) causes me a feeling of internal discomfort.
  - The constant presence of air conditioning causes me a feeling of internal discomfort.
  - I feel that the workplace equipment (chairs, desks, monitors, computer control devices) does not suit my needs.
  - During my work, I enter areas with an active radiation source (for example, fluoroscopic examinations, intraoperative radiography, or interventional radiology procedures).
  - I feel that the measures for my radiation protection, including personal protective equipment, are insufficient.

The first four items are answered by a frequency Likert scale. The last three questions are dichotomous.

3. **Professional isolation and patient-related feedback** (2 items)
  - I lack information about the outcomes of treatment of patients whose diagnosis or treatment I contributed to.
  - I feel more like a “service provider” for other clinical specialties rather than an equal partner in the treatment process.

All of these are answered by a frequency Likert scale.

4. **Lifestyle-related occupational stressors** (4 items)
  - On average, how many 24-h on-call shifts do you work per month?
  - On average, how much time per week do you spend using screen-based devices? (Including smartphones and gaming consoles).
  - If possible, please provide your smartphone “screen time” data. (Optional question).
  - I feel that stress and excessive workload lead to the following forms of maladaptive coping/overuse behavior. (Multiple responses may be selected; this is a subjective self-assessment.) Options: overeating, excessive coffee consumption, smoking. These were data-entry questions.

In all, 9 questions were measured using a 5-point Likert scale, 3 were dichotomous (yes/no), and 4 required data input. The instrument was not formally pilot-tested prior to deployment. The complete English translation of the questionnaire is provided in Supplementary Material S1, the full questionnaire used in Slovak is provided in Supplementary Material S2.

### 2.3. Study Population and Data Collection

Participants were selected using convenience sampling. The questionnaire was distributed by selected colleagues and heads of departments. A total of 213 questionnaires were distributed among radiology departments and workplaces across Slovakia using both paper-based and electronic formats. 107 questionnaires were returned for a return rate of approximately 50.2%. Incomplete questionnaires or those missing essential BAT items or key demographic variables were excluded from analysis. In all two paper-based questionnaires were excluded. A total of 105 fully completed questionnaires were included in the final analysis, yielding a valid response rate of 49.3%.

The study included participants from multiple healthcare institutions across different Slovak regions. Participants were recruited from large hospitals, smaller regional hospitals, specialized centers and private clinics ensuring geographic and institutional diversity. Participation was voluntary; questionnaires were filled anonymously online or send anonymously via mail. All respondents provided informed consent.

### 2.4. Inclusion and Exclusion Criteria

#### 2.4.1. Inclusion Criteria

- Employment in a radiology department or radiology workplace in Slovakia
- Age ≥ 18 years
- Occupation as radiologist, radiology technician/radiographer, nurse, or administrative staff
- Voluntary informed consent

#### 2.4.2. Exclusion Criteria

- Incomplete BAT responses preventing score computation
- Duplicate responses
- Employment outside radiology practice or outside Slovakia
- Withdrawal of consent

### 2.5. Statistical Analysis

Data were analyzed using **Microsoft Excel** and **IBM SPSS Statistics (version 26)**. Prior to analysis, data were screened for completeness, consistency, and distributional assumptions.

#### Descriptive Statistics

Continuous variables (BAT scores and subscale scores) were expressed as **mean ± standard deviation (SD)**. Categorical variables were presented as **frequencies and percentages**. Normality of distribution was assessed using the **Shapiro–Wilk test**.

### 2.6. BAT Scoring Procedure

The Burnout Assessment Tool (BAT) questionnaire was scored according to the methodology described by Schaufeli et al. [20]. Individual item responses were rated using a Likert scale: 1 (“never”), 2 (“rarely”), 3 (“sometimes”), 4 (“often”) and 5 (“always”).

Scores for the four core dimensions of burnout—exhaustion, mental distance, cognitive impairment, and emotional impairment- were calculated as the arithmetic mean of the corresponding items within each dimension. The overall BAT score was calculated as the mean value of all core BAT items. Higher scores indicated a higher level of burnout symptoms and higher risk of burnout syndrome. Since country specific cutoff values are not available interpretation of the BAT scores was based on published general cut-off values [20]: Overall BAT scores between **1.00–2.58** indicated low risk of burnout, scores between **2.59–3.01** indicated elevated risk, and scores of **3.02** and above indicated high risk. Secondary symptoms (psychological distress, psychosomatic complaints) were analyzed separately and reported as individual domain scores. The interpretation was based on general scale separate for every dimension [20]. The BAT core symptoms cut-offs are presented in Table 1, the secondary symptoms cut-offs in Table 2.

#### 2.6.1. Reliability Analysis

Internal consistency of BAT scores was assessed using Cronbach’s alpha (α).

#### 2.6.2. Correlation Analysis

Associations between continuous variables (e.g., number of 24-h on-call shifts and BAT scores) were assessed using:

- Spearman Rho rank correlation.

Statistical significance was defined as *p* < 0.05.

#### 2.6.3. Group Comparisons

Comparisons between two groups were performed using the Mann-Whitney test.

### 2.7. Missing Data Handling

Only fully completed BAT questionnaires were included in the final analysis. Two paper based questionnaires were received incomplete with majority of sheets missing. No imputation of missing values was performed.

### 2.8. Ethical Considerations

The study was approved by the Ethics Review Committee of the central military hospital in Ružomberok, Slovakia (UVN SNP Ružomberok—FN), approval No. ÚVN 18-48/2025, approved on 16 June 2025. Before participation, all potential participants received a written participant information sheet describing the study objectives, voluntary nature of participation, expected completion time, confidentiality measures, data processing procedures, and the absence of foreseeable risks associated with participation. Following the information sheet, participants were asked to provide explicit informed consent by answering the question, “I agree to participate in this research study” (Yes/No). Only respondents who selected “Yes” were able to proceed to the questionnaire. Participants could decline participation by selecting “No” or by discontinuing the questionnaire at any time without providing a reason.

The questionnaire was anonymous. No names or other direct personal identifiers were collected, and all data were analyzed in aggregate form. Distribution of the questionnaire through participating radiology departments did not allow department heads or employers to identify individual participants or their responses.

## 3. Results

### 3.1. Participant Characteristics

The final study sample consisted of 105 respondents working in radiology healthcare settings in Slovakia. The majority of participants were female (*n* = 67; 63.8%), while males accounted for 36.2% of the sample (*n* = 38). Regarding age distribution, the largest proportion of respondents was in the 31–40-year age group (*n* = 38; 36.2%), followed by participants aged ≤ 30 years (*n* = 28; 26.7%). Participants aged 41–50 years and ≥51 years represented 19.0% (*n* = 20) and 18.1% (*n* = 19) of the sample, respectively.

In terms of professional experience, the distribution was relatively balanced across categories, with the largest groups consisting of respondents with 10–15 years of healthcare experience (*n* = 21; 20.0%) and 4–6 years of experience (*n* = 19; 18.1%). The occupational composition of the sample included physicians/radiologists (*n* = 46; 43.8%), radiology technicians (*n* = 46; 43.8%), nurses (*n* = 8; 7.6%), and administrative staff (*n* = 5; 4.8%). Detailed demographic and professional characteristics of the study participants are presented in Table 3.

### 3.2. Burnout Assessment Tool Results

The Cronbach alpha of the 33 item BAT questionnaires results with *N* = 105 has been calculated as α = 0.93. The dominant domain of burnout syndrome in the set of respondents was exhaustion (3.06) with standard deviation (SD) 0.65, specific was a relatively high level of cognitive impairment (2.76) with standard deviation 0.76, the mental distance was calculated at 2.21 with standard deviation 0.82 and emotional impairment was calculated at 2.16 with standard deviation 0.72. The secondary symptoms of psychological distress were calculated at 2.65 with standard deviation 0.85 and the secondary symptoms of psychosomatic complaints were calculated at 2.34 with standard deviation 0.67. On the other hand, emotional impairment (2.16, SD 0.72) and mental distancing (2.21, SD 0.82) were lower. 24.76% (*N* = 26) of respondents had overall BAT value at 3.02 or above and can be considered in high risk for burnout. 61.9% (*N* = 65) of respondents had at least one burnout dimension elevated to high risk. The summary of BAT average scores is in Table 4.

Because of the fact that the sample (*N* = 105) shows non-normal distribution we decided to use the Spearman’s Rho (ρ) Rank Correlation to assess the relationship between the number of 24 shifts and the level of mental distancing. We only included radiology technicians and radiologists in the calculation, since nurses and administrative staff in radiology setting don’t usually work 24 h shifts. The average number of 24 h shifts worked by the radiologist was M = 3.41. The average number of 24 h shifts worked by the radiology technicians was M = 2.70.

Group A (0, 1, 2 shifts): The average of mental distance 1.69 (*N* = 38)

Group B (3, 4 shifts): The average of mental distance 2.17 (*N* = 24)

Group C (5 and more shifts): The average of mental distance 3.05 (*N* = 30)

To represent the groups averages of 24 shifts were used (1 for group A, 3.5 for group B and 6 for group C).

Spearman’s ρ = 0.65, *p* < 0.001, *N* = 92

Additionally, in this exploratory analysis, radiologists aged 40 years or younger who reported working five or more 24-h shifts per month (*N* = 11) exhibited a mean cognitive impairment score of 3.24. This observation suggests a pattern of elevated cognitive impairment within this subgroup, although the small sample size warrants cautious interpretation.

The level of cognitive impairment dimension was compared in the subsets of doctors and radiology technicians. Both groups consist of 46 respondents. Normality test for radiology technicians—The Shapiro-Wilk test showed a significant departure from normality, W(46) = 0.94, *p* = 0.022. Normality test for radiologist—The Shapiro-Wilk test did not show a significant departure from normality, W(46) = 0.96, *p* = 0.093. Due to the results of the normality tests Mann-Whitney test was used to test whether the difference is significant with the result: U = 495.50, *p* < 0.001. The summary of the results is in Table 5.

### 3.3. Author-Developed Exploratory Instrument Results

As part of the exploratory descriptive analysis, the author-developed exploratory instrument identified two notable patterns. Among radiologists (*N* = 46), the highest mean score was observed for visual exhaustion (Question 5), with a mean Likert score of 3.78 (SD = 0.99; 95% CI: 3.49–4.07). Across the entire study sample (*N* = 105), professional isolation (Questions 11 and 12) showed a mean Likert score of 3.40 (SD = 1.15; 95% CI: 3.18–3.62). Overview of these findings is in Table 6.

These findings represent descriptive observations intended to characterize potential occupational stressors in the study population. Table 6 presents the mean Likert scores and standard deviations for visual exhaustion among radiologists and professional isolation across the entire sample. Because the psychometric properties of the author-developed exploratory instrument have not yet been formally established, these findings should be interpreted as preliminary and hypothesis-generating rather than confirmatory.

## 4. Discussion

The first objective of our study was to quantify the main dimensions of burnout (exhaustion, mental distance, cognitive impairment, emotional impairment) using the Burnout Assessment Tool (BAT) in employees of radiology departments and clinics. Our findings indicate clinically relevant levels of burnout, with the highest mean scores observed for exhaustion (M = 3.06) and cognitive impairment (M = 2.76). These results suggest that radiology work is likely predominantly burdened by mental exhaustion and a heightened risk of cognitive failure, which may be less typical in some other medical specialties but can be critical in a cognitively demanding, visually intensive specialty and has been linked to interpretive error and reduced diagnostic accuracy [7,14,21]. Reviews of interpretive error emphasize that radiologists work under “increasing workloads and fatigue,” with error rates of ~3–5% in daily practice and retrospective error around 30% [22,23]. Overall, 24.76% of respondents had an overall BAT value 3.02 or above, indicating a risk of burnout, which is in line with European radiology data showing high burnout prevalence [7,24]. In addition, 61.9% of respondents had at least one BAT dimension at high risk level, consistent with international findings that a majority of radiologists report at least one elevated burnout domain [7,13,25,26,27].

### 4.1. Based on the BAT Questionnaire, We Established a Profile of All Four Core Symptoms of Burnout

Exhaustion (M = 3.06): This was the highest value in our set, indicating chronic overburden, which may be due to high number of 24 h shifts. High exhaustion as the central burnout symptom and main discriminator of severe burnout has been repeatedly demonstrated in BAT studies and physician burnout research [7,13,14,24].

Mental distance (M = 2.21): The overall average is at a low risk level; however, in the group with ≥5 monthly 24-h emergency shifts it is significantly elevated to (M = 3.05), which suggests the 24 h shifts may contribute in an important way to this specific symptom. This indicates that overload with 24-h shifts may lead to mental distancing, in line with BAT cut-off work and health-professional data where mental distance increases with chronic demands and insufficient recovery [11,17,18,26]. Similar patterns are reported for physicians and non-consultant doctors in mixed-shift systems, where extended and night work are associated with detachment, reduced well-being, and burnout [7,13,14,23,25].

Cognitive impairment (M = 2.76): The second-highest average among the dimensions. The elevated level points to the specifics of radiology, which requires extreme mental burden when analyzing visual data, and may be associated with the risk of diagnostic error. BAT validation studies and health-professional samples show that cognitive impairment is a key burnout manifestation and is linked to functional problems in attention and memory [7,23,25]. In radiology, cognitive overload, fatigue, and high workload have been empirically linked to perceptual misses, interpretive errors, and increased malpractice risk [7,21,23].

Emotional impairment (M = 2.16): This level is the lowest in our set. This may suggest that burnout in radiology does not primarily manifest as emotional exhaustion from patient contact (considered common in nurses or caregivers), but may be driven mainly by mental and technological stressors. BAT-based studies also show that emotional impairment may be less dominant than exhaustion and cognitive impairment in some professional groups, while still contributing to interpersonal strain over time [7,23,26].

### 4.2. International Context and Occupational Factors Associated with Burnout

Comparison of burnout findings between countries requires caution due to substantial methodological heterogeneity among studies. Previous investigations have used different assessment instruments, including the Maslach Burnout Inventory (MBI) and the Burnout Assessment Tool (BAT), applied different thresholds for defining burnout, and included heterogeneous professional groups and study periods. Therefore, reported burnout prevalence rates should not be considered directly comparable. Instead, international studies provide insight into common occupational factors associated with burnout among radiology professionals. Selected international studies and their methodological characteristics are summarized in Table 7.

Several studies indicate that burnout symptoms among radiologists increased during and after the COVID-19 pandemic, although differences in methodology limit direct comparisons. A Spanish study conducted among 150 radiologists during the COVID-19 pandemic reported burnout risk in 49.3% of participants, while a comparable pre-pandemic study reported a lower prevalence (33.6%). Similarly, increased burnout levels have been described in other healthcare systems following periods of intensified workload and organizational pressure [28]. In the present study, 61.9% of respondents demonstrated elevation in at least one BAT dimension above the established high risk threshold, while 24.76% reached the threshold for high overall burnout risk. These findings indicate substantial burnout-related symptoms in the studied population; however, they should not be interpreted as directly equivalent to prevalence estimates reported using other instruments.

Across different countries, workload-related factors appear to represent a consistent contributor to burnout among radiology professionals. Studies from the United States, Canada, Germany, and other healthcare systems have reported high levels of burnout symptoms among radiologists, often associated with increasing imaging volumes, staffing shortages, on-call responsibilities, and growing professional demands [24,29,31]. Similarly, studies among interventional radiologists in the United Kingdom have demonstrated substantial levels of emotional exhaustion, further highlighting the influence of occupational demands on physician well-being [33].

Differences between professional groups may also contribute to variation in reported burnout rates. Studies including radiographers, sonographers, and radiologists have demonstrated burnout symptoms across all occupational categories, although the nature of occupational stressors may differ according to professional responsibilities [32]. In the present study, radiologists demonstrated higher cognitive impairment scores compared with radiology technicians, suggesting that sustained diagnostic responsibility and cognitive workload may represent important contributors to burnout symptoms.

The cognitive impairment observed in our study (mean BAT score 2.76) may reflect several occupational challenges present in radiology practice, including employee shortages, increased workload, technological demands, and frequent 24-h shifts. These factors are consistent with international findings demonstrating associations between workload intensity, sleep disruption, working conditions, and burnout symptoms among radiology professionals [24,25].

Longitudinal monitoring of burnout among radiology professionals would provide more reliable information regarding temporal trends. Although repeated national surveys have been performed in some countries, such as the United States, comparable systematic monitoring has not yet been established in Slovakia [34]. Future studies using standardized instruments and consistent methodology are needed to evaluate changes in burnout levels among Slovak radiology healthcare workers.

The level of cognitive impairment we observed (2.76) may also reflect employee shortages, overburden, and frequent 24-h shifts in radiology clinics, which would be consistent with findings from studies on radiologists in Germany, Canada, and other countries where workload, sleep problems, and poor working conditions are strongly associated with burnout [24,25]. Again relevant studies in Slovakia have not been done yet.

### 4.3. Association Between Shift Work and Burnout Symptoms

The expected increase of burnout symptoms with increasing numbers of 24-h shifts was present in our set and manifested mainly in doctors, especially those younger than 40 years, with ≥5 24-h shifts per month (cognitive impairment in this subgroup 3.24). The dominant burnout dimension in this high-shift group is mental distance (3.05), consistent with evidence that exhaustion and emotional or cognitive impairment, combined with low social support, predict later interpersonal strain and withdrawal at work [7,8,11]. This is also in line with systematic reviews on mixed shift rotations that link extended hours and night work to sleep disruption, fatigue, burnout, and impaired well-being [8,11,14,15,16]. In the subgroup of younger radiologists under 40 years of age working at least five 24-h shifts per month, cognitive impairment reached 3.24.

### 4.4. Differences Between Radiologists and Radiology Technicians

The comparison of burnout symptoms between radiologists and radiology technicians suggests that doctors experience more cognitive impairment, probably due to the large volume of complex visual data they must process and the decisional responsibility for final reports. Technicians’ tasks may require somewhat less sustained high-level cognitive processing and include more direct patient contact, which can mitigate feelings of depersonalization and isolation despite high workload. Professional isolation, often described in radiology because of working in dark reading rooms with limited direct patient and colleague contact, likely is an independent stressor linked to burnout and was reported as a frequent experience in our questionnaire, echoing international observations of isolation and “service provider” identity as contributors to radiologist burnout.

### 4.5. Visual Exhaustion and Occupational Demands

The elevated level of visual exhaustion among radiologists identified using the exploratory instrument (mean Likert score 3.78) may reflect the increasing visual demands associated with prolonged diagnostic image interpretation. Similar concerns regarding visual strain among radiologists have been reported internationally, with digital eye strain and prolonged monitor-based work identified as potential contributors to occupational stress and diagnostic fatigue [35]. Previous studies into visual strain suffered by radiologists showed less strain [36]. This may be caused by rapid shift from analog radiology into digital and the accompanied increase in workload. The studies performed during and after the COVID era show a significant increase [37]. The importance of optimal workstation ergonomics and optimal lighting conditions has been shown [38].

### 4.6. Study Limitations

The limited number of respondents was one of the major limitations of our research. Although many hospitals and workplaces were contacted and 213 questionnaires were distributed, fewer than 50% were returned in valid state. This may be due to the length of the full questionnaire (55 questions), which could have been perceived as burdensome and discouraged participation, a challenge also noted in other burnout survey studies [24,28,30,31]. Additionally, non-response bias cannot be excluded, as healthcare workers experiencing higher levels of exhaustion or burnout-related symptoms may have been either more or less likely to participate. Notably only a few nurses and administrative staff participated, which is a major limitation. Further serious limitations are cross-sectional study design, convenience sampling, non-validation of the exploratory instrument and lack of multivariable adjustment. There are also no specific Slovak cut-off values for BAT scoring, which may lead to bias. Another limitation is that some respondents may have focused more on their current psychological state than on long-term trends in their professional life, a known issue in cross-sectional, self-report burnout research. Furthermore, due to convenience sampling, relatively low response rate, very low number of nurses and administrative staff and the inclusion of only selected regions of Slovakia our sample cannot be considered representative for Slovakia, which underlines the need for larger, longitudinal, and broader multi-center studies using the BAT in radiology to draw any serious conclusions and infer causality in any of the factors that may contribute to burnout. In order to reach firm decisions on any preventive measures more robust and focused research is necessary.

## 5. Conclusions

Radiology was historically perceived as a relatively low-stress medical specialty; however, with the advent of digitization and PACS, the workload in radiology departments has been steadily increasing. This sustained increase in workload is accompanied by growing pressure and stress, particularly among radiologists.

Our research suggests that approximately 24.76% of employees in radiology clinics and departments in Slovakia may be at risk of developing burnout syndrome and may already be experiencing its symptoms. The burnout profile among radiology staff in our set is dominated by the dimensions of exhaustion and cognitive impairment. The level of cognitive impairment reported by our respondents requires further robust and focused research.

We further found that an increasing number of 24-h shifts may be associated with an increase in mental distance. In our sample at five or more 24-h shifts per month mental distance average reached M = 3.05, signaling elevated risk. Further focused research is necessary to clarify the situation.

The finding of higher cognitive impairment in radiologist compared to radiology technicians suggests the occupations suffer from different types or levels of strain. This suggests these positions may require individual approach to management of workforce. The findings of elevated levels of professional isolation and visual exhaustion point to specific factors that may be contributing to the risk of burnout associated with the radiology workplace. These factors must be further explored with better tools and a larger sample to confirm the relationships. There are studies, which suggest measures such as scheduled micro-breaks (20-20-20 rule), regular eye exams, optimized lighting (low level ambient lighting), optimal viewing distance and high-quality (diagnostic grade) displays are evidence-based strategies to mitigate eyestrain and protect visual function in radiology staff, with frequent short breaks showing the clearest association with lower digital eye strain [36].

Given the mounting pressure and the steadily increasing volume of imaging examinations, including in smaller hospitals and sparsely populated regions, active management of workflow in radiology departments is becoming essential to maintain diagnostic accuracy and optimize patient outcomes. Engagement from leadership at all organizational levels is needed not only to preserve the health and effectiveness of staff, but also to retain them, especially in the context of competing opportunities in smaller private clinics and telemedicine. Finally, staff turnover appears to be an additional challenge that may be related to burnout and deserves further systematic investigation. Our study hints at a specific dimension profile of burnout risk in radiology staff of Slovakia, with specific factors shown to warrant further research to devise measures to improve the effectiveness of radiology clinics and departments.

## Figures and Tables

**Table 1:** BAT 23 subscale cut-offs.

Scale	Low Risk	Intermediate Risk	Elevated Risk
Exhaustion	1.00–3.05	3.06–3.30	3.31–5.00
Mental Distance	1.00–2.49	2.50–3.09	3.10–5.00
Cognitive Impairment	1.00–2.69	2.70–3.09	3.10–5.00
Emotional Impairment	1.00–2.09	2.10–2.89	2.90–5.00

**Table 2:** Cut-offs for the secondary symptoms.

Secondary Symptoms Score	Interpretation
1.00–2.84	Green
2.85–3.34	Orange
3.35–5.00	Red

**Table 3:** Demographic and professional characteristics of study participants (*N* = 105).

Characteristic	*N*	%
**Sex**		
Male	38	36.2
Female	67	63.8
**Age group (years)**		
≤30	28	26.7
31–40	38	36.2
41–50	20	19.0
≥51	19	18.1
**Years of experience in healthcare**		
1–3 years	16	15.2
4–6 years	19	18.1
7–9 years	13	12.4
10–15 years	21	20.0
16–25 years	17	16.2
≥26 years	19	18.1
**Professional position**		
Physicians/radiologists	46	43.8
Radiology technicians	46	43.8
Nurses	8	7.6
Administrative staff	5	4.8

**Table 4:** Average score of BAT dimensions + Overall BAT scores, Standard deviation, Confidence interval and interpretation. For every domain (*N* = 105).

	BAT Score Average	Standard Deviation	Confidence Interval	Interpretation
CS—exhaustion	3.06	0.65	95% CI, 2.94–3.18	Elevated risk
CS—mental distance	2.21	0.82	95% CI, 2.05–2.37	Low risk
CS—cognitive impairment	2.76	0.76	95% CI, 2.62–2.91	Elevated risk
CS—emotional impairment	2.16	0.72	95% CI, 2.02–2.30	Elevated risk
SS—psychological distress	2.65	0.85	95% CI, 2.49–2.81	Low risk
SS—psychosomatic complaints	2.34	0.67	95% CI, 2.21–2.47	Low risk
OBS—core symptoms	2.61	0.53	95% CI, 2.51–2.71	Elevated risk
OBS—secondary symptoms	2.49	0.67	95% CI, 2.36–2.62	Low risk

**Table 5:** Level of cognitive impairment of Doctors and Radiology technicians.

Profession	Respondents (*N*)	Average (M)	Standard Deviation (SD)	Mann-Whit-Ney Test (U)	Confidence Interval
Radiologist (doctor)	46	3.12	0.80	U = 495.50, *p* < 0.001	95% CI: 2.89–3.35
Radiology technician (radiographer)	46	2.42	0.58	U = 495.50, *p* < 0.001	95% CI: 2.25–2.59

**Table 6:** Exploratory descriptive findings from the author-developed occupational stressor instrument.

Occupational Stressor	Population Assessed	Item(s)	*N*	M (Likert Score)	SD	95% CI
Visual exhaustion	Radiologists	Question 5	46	3.78	0.99	3.49–4.07
Professional isolation	All participants	Questions 11–12	105	3.40	1.15	3.18–3.62

**Table 7:** Selected international studies investigating burnout among radiology professionals and methodological characteristics.

Study	Country	Population	Year	Instrument	Main Reported Finding	Relevant Occupational Factors
Oprisan et al. [28]	Spain	Radiologists (*N* = 150)	2020	MBI	Burnout risk: 49.3% during COVID-19; 33.6% pre-pandemic	Increased workload during pandemic period
Parikh et al. [29]	USA	Private practice radiologists (*N* = 40)	2022	MBI	Burnout reported in 33%	Private practice workload and organizational factors
Bin Dahmash et al. [30]	Saudi Arabia	Radiologists (*N* = 108)	2020	MBI	High burnout risk: 24.1%	Occupational stressors among radiologists
Zha et al. [31]	Canada	Radiologists (*N* = 262)	2018	MBI	Single-domain burnout: 72%	Workload and professional demands
Singh et al. [32]	Australia/New Zealand	Radiologists, radiographers, sonographers	2017	MBI	Single-domain burnout: radiologists 44.9%; radiographers 39.9%; sonographers 42.2%	Differences between professional roles
Bastian et al. [24]	Germany	Radiologists (*N* = 172)	2024	MBI	Burnout reported in 76.7%	Workload and workplace conditions
Al Rekabi et al. [33]	UK	Interventional radiologists (*N* = 223)	2023	MBI	Moderate/severe emotional exhaustion: 65%	Procedural workload and occupational demands
Present study	Slovakia	Radiology healthcare workers (*N* = 105)	—	BAT-33	High-risk overall burnout: 24.8%; ≥1, elevated BAT dimension: 61.9%	Shift work, cognitive workload, occupational stressors

## Data Availability

The data presented in this study are available on request from the corresponding author.

## Supplementary Materials

The following supporting information can be downloaded at: https://www.mdpi.com/article/10.3390/healthcare14152385/s1, Supplementary Material S1: English Translation of the Study Questionnaire, Supplementary Material S2. Original Slovak Version of the Study Questionnaire and Informed Consent.
