# Supplementary material for: Burnout and Associated Risk Factors a Cross-Sectional Study Among Staff of Radiology Clinics in Slovakia
Source: Healthcare (Basel). 2026 Aug 4;14(15):2385. doi: 10.3390/healthcare14152385 (PMC13465215; doi:10.3390/healthcare14152385)
Supplement: Supplementary file 1 [file healthcare-14-02385-s001.zip › Supplementary Material S1.pdf]

Supplementary Material S1. English Translation of the Study Questionnaire

General questions

The general questions have been translated into English and are as follows:

1. I consent to participate in this research study.
2. Sex:
3. Age:
4. Years of experience in healthcare:
5. Occupation:
6. Highest educational attainment:

BAT - Work-Related version

Exhaustion

1. At work, I feel mentally exhausted*
2. Everything I do at work requires a great deal of effort
3. After a day at work, I find it hard to recover my energy*
4. At work, I feel physically exhausted*
5. When I get up in the morning, I lack the energy to start a new day at work
6. I want to be active at work, but somehow I am unable to manage
7. When I exert myself at work, I quickly get tired
8. At the end of my working day, I feel mentally exhausted and drained

Mental Distance

9. I struggle to find any enthusiasm for my work*
10. At work, I do not think much about what I am doing and I function on autopilot*
11. I feel a strong aversion toward my job
12. I feel indifferent about my job
13. I am cynical about what my work means to others*

Cognitive Impairment

14. At work, I have trouble staying focused*
15. At work, I struggle to think clearly
16. I'm forgetful and distracted at work
17. When I'm working, I have trouble concentrating*
18. I make mistakes in my work because I have my mind on other things*

Emotional Impairment

19. At work, I feel unable to control my emotions*
20. I do not recognize myself in the way I react emotionally at work*
21. During my work, I become irritable when things don't go my way
22. I get upset or sad at work without knowing why
23. At work, I may overreact unintentionally

Psychological Complaints
24. I have trouble falling or staying asleep
25. I tend to worry
26. I feel tense and stressed
27. I feel anxious and/or suffer from panic attacks
28. Noise and crowds disturb me

Psychosomatic Complaints
29. I suffer from palpitations or chest pain
30. I suffer from stomach and/or intestinal complaints
31. I suffer from headaches
32. I suffer from muscle pain, for example in the neck, shoulder or back
33. I often get sick

Author-Designed questionnaire

The questions of the author designed questionnaire have been translated into English and are as follows:

This set of questions is answered with a five-point frequency Likert scale:
1. I experience anxiety or tension when new versions of diagnostic software (PACS/RIS) and new imaging equipment are introduced.
2. I perceive the number of artifacts and technical errors during examinations as a frustrating factor that slows down my work.
3. I feel that expectations regarding my work speed are increasing faster than the performance of the technologies I use.
4. Working in an environment with insufficient natural daylight negatively affects my mood and energy levels.
5. During my work shift, I experience significant visual fatigue (eye strain, burning eyes, blurred vision) as a result of prolonged monitor use.
6. Noise from imaging equipment (especially MRI scanners) causes me a feeling of internal discomfort.
7. The constant presence of air conditioning causes me a feeling of internal discomfort.

This set of questions consists of yes/no items:
8. I feel that the workplace equipment (chairs, desks, monitors, computer control devices) does not suit my needs.
9. During my work, I enter areas with an active radiation source (for example, fluoroscopic examinations, intraoperative radiography, or interventional radiology procedures).
10. I feel that the measures for my radiation protection, including personal protective equipment, are insufficient.

This set of questions is answered with a five-point frequency Likert scale:
11. I lack information about the outcomes of treatment of patients whose diagnosis or treatment I contributed to.
12. I feel more like a "service provider" for other clinical specialties rather than an equal partner in the treatment process.

This set of questions requires data entry:

13. On average, how many 24-hour on-call shifts do you work per month?

14. On average, how much time per week do you spend using screen-based devices? (including smartphones and gaming consoles).

15. If possible, please provide your smartphone "screen time" data. (Optional question).

16. I feel that stress and excessive workload lead to the following forms of maladaptive coping/overuse behavior. (Multiple responses may be selected; this is a subjective self-assessment.) Options: overeating, excessive coffee consumption, smoking.
