# Supplementary material for: Burnout and Associated Risk Factors a Cross-Sectional Study Among Staff of Radiology Clinics in Slovakia
Source: Healthcare (Basel). 2026 Aug 4;14(15):2385. doi: 10.3390/healthcare14152385 (PMC13465215; doi:10.3390/healthcare14152385)
Supplement: Supplementary file 1 [file healthcare-14-02385-s001.zip › Supplementary Material S2.pdf]

Supplementary Material S2. Original Slovak Version of the Study Questionnaire and Informed Consent

Diagnostický nástroj na meranie BURN-OUT syndrómu – BAT

INFORMOVANÝ SÚHLAS S ÚČASŤOU VO VÝSKUME

Názov výskumu: **Prevencia syndrómu vyhorenia pracovníkov v zdravotníctve**

Výskumník: MUDr. Michal Slašťan MPH

Inštitúcia: Vysoká škola zdravotníctva a sociálnej práce sv. Alžbety

Vážená pani / Vážený pán, obraciam sa na Vás s prosbou o účasť vo výskume, ktorý je súčasťou mojej dizertačnej práce. Cieľom tohto výskumu je zmapovať mieru psychickej záťaže a prejavy vyhorenia v prostredí zdravotníctva.

Informácie pre účastníka:

- Dobrovoľnosť: Účasť na výskume je úplne dobrovoľná. Svoju účasť môžete kedykoľvek prerušiť bez uvedenia dôvodu.
- Anonymita a ochrana dát: Výskum je anonymný. Získané údaje budú spracované hromadne a použité výhradne na účely tejto akademickej práce. Vaše meno ani iné priame identifikačné údaje sa nikde neuvádzajú.
- Časová náročnosť: Vyplnenie dotazníka vám zaberie približne 10 – 15 minút.
- Riziká a prínosy: Účasť vo výskume nepredstavuje žiadne riziko. Vaše odpovede však môžu prispieť k lepšiemu pochopeniu pracovnej záťaže v zdravotníctve.

Súhlas účastníka: Pokračovaním v dotazníku potvrdzujete, že ste boli informovaný/á o cieľoch výskumu a súhlasíte s dobrovoľným spracovaním svojich odpovedí na účely akademického spracovania.

Inštrukcie:

Výskum je zameraný na pracovné zaťaženie pracovníkov v medicínskom odbore rádiológia.

Nasledujúce tvrdenia sa týkajú vašej pracovnej situácie a toho, ako túto situáciu prežívate. Uveďte, prosím, ako často sa vás jednotlivé výroky týkajú.

* Označuje povinnú otázku

1. *Súhlasím s účasťou vo výskume* *

Začiarknite vyhovujúcu možnosť

☐ Áno
☐ Nie

Všeobecné otázky

Prosím pravdivo odpovedajte na otázky o vašej osobe.

2. Pohlavie *

Označte iba jednu elipsu.

◯ Muž
◯ Žena

3. Vek *

Označte iba jednu elipsu.

◯ Do 30 rokov
◯ 31 - 40 rokov
◯ 41 - 50 rokov
◯ 51 a viac rokov

4. Počet rokov odpracovaných v zdravotníctve *

Označte iba jednu elipsu.

- 1 - 3 roky
- 4 - 6 rokov
- 7 - 9 rokov
- 10 - 15 rokov
- 16 - 25 rokov
- 26 a viac rokov

5. Pracovná pozícia *

Označte iba jednu elipsu.

- Sestra
- Lekár
- Rádiologický technik
- Technicko-administratívny pracovník

6. Najvyššie ukončené vzdelanie *

Označte iba jednu elipsu.

- Základné
- Stredoškolské bez maturity
- Stredoškolské s maturitou
- Vysokoškolské I. stupňa
- Vysokoškolské II. stupňa
- Vysokoškolské III. stupňa

Základné/jadrové symptómy

Vyčerpanosť.

7. V práci sa cítim psychicky vyčerpaný/á. *

Označte iba jednu elipsu.

- Nikdy
- Zriedka
- Občas
- Často
- Vždy

8. Všetko, čo v práci robím si vyžaduje veľké množstvo úsilia. *

Označte iba jednu elipsu.

- Nikdy
- Zriedka
- Občas
- Často
- Vždy

9. Po celom dni v práci je pre mňa ťažké získať späť svoju energiu. *

Označte iba jednu elipsu.

- Nikdy
- Zriedka
- Občas
- Často
- Vždy

10. V práci sa cítim fyzicky vyčerpaný/á. *

Označte iba jednu elipsu.

- Nikdy
- Zriedka
- Občas
- Často
- Vždy

11. Keď ráno vstávam, chýba mi energia začať nový pracovný deň. *

Označte iba jednu elipsu.

- Nikdy
- Zriedka
- Občas
- Často
- Vždy

12. Chcem byť v práci aktívny/a, ale nejako sa mi to nedarí. *

Označte iba jednu elipsu.

- Nikdy
- Zriedka
- Občas
- Často
- Vždy

13. Keď sa v práci namáham, rýchlo sa unavím. *

Označte iba jednu elipsu.

- Nikdy
- Zriedka
- Občas
- Často
- Vždy

14. Na konci svojho pracovného dňa sa cítim psychicky vyčerpaný/á a vyšťavený/á. *

Označte iba jednu elipsu.

- Nikdy
- Zriedka
- Občas
- Často
- Vždy

Psychický odstup

15. Ťažko sa mi hľadá nadšenie pre svoju prácu. *

Označte iba jednu elipsu.

- Nikdy
- Zriedka
- Občas
- Často
- Vždy

16. V práci veľmi nepremýšľam nad tým, čo robím a fungujem na autopilota. *

Označte iba jednu elipsu.

- Nikdy
- Zriedka
- Občas
- Často
- Vždy

17. Cítim silný odpor voči svojej práci. *

Označte iba jednu elipsu.

- Nikdy
- Zriedka
- Občas
- Často
- Vždy

18. Cítim ľahostajnosť voči svojej práci. *

Označte iba jednu elipsu.

- Nikdy
- Zriedka
- Občas
- Často
- Vždy

19. Som cynický/á, pokiaľ ide o význam mojej práce pre ostatných. *

Označte iba jednu elipsu.

- Nikdy
- Zriedka
- Občas
- Často
- Vždy

Kognitívne problémy

20. V práci mám ťažkosti s udržaním sústredenia. *

Označte iba jednu elipsu.

- Nikdy
- Zriedka
- Občas
- Často
- Vždy

21. V práci je pre mňa ťažké uvažovať jasne. *

Označte iba jednu elipsu.

- Nikdy
- Zriedka
- Občas
- Často
- Vždy

22. V práci som zábudlivý/á a roztržitý/á. *

Označte iba jednu elipsu.

- Nikdy
- Zriedka
- Občas
- Často
- Vždy

23. Keď pracujem, mám problém sústrediť sa. *

Označte iba jednu elipsu.

- Nikdy
- Zriedka
- Občas
- Často
- Vždy

24. V práci robím chyby, pretože myslím na iné veci. *

Označte iba jednu elipsu.

- Nikdy
- Zriedka
- Občas
- Často
- Vždy

Oslabenie emočnej kontroly

25. V práci mám pocit, že nedokážem ovládať svoje emócie. *

Označte iba jednu elipsu.

- Nikdy
- Zriedka
- Občas
- Často
- Vždy

26. Nespoznávam sa, ako emocionálne reagujem v práci. *

Označte iba jednu elipsu.

- Nikdy
- Zriedka
- Občas
- Často
- Vždy

27. Som podráždený/á, keď sa mi nedarí počas práce. *

Označte iba jednu elipsu.

- Nikdy
- Zriedka
- Občas
- Často
- Vždy

28. V práci som rozrušený/á alebo smutný/á a neviem prečo. *

Označte iba jednu elipsu.

- Nikdy
- Zriedka
- Občas
- Často
- Vždy

29. V práci sa mi stáva, že neúmyselne prehnane reagujem. *

Označte iba jednu elipsu.

- Nikdy
- Zriedka
- Občas
- Často
- Vždy

Sekundárne symptómy

Psychické ťažkosti

30. Mám ťažkosti so zaspávaním alebo zotrvaním v spánku. *

Označte iba jednu elipsu.

- Nikdy
- Zriedka
- Občas
- Často
- Vždy

31. Mám sklony k obavám. *

Označte iba jednu elipsu.

- Nikdy
- Zriedka
- Občas
- Často
- Vždy

32. Cítim napätie alebo stres. *

Označte iba jednu elipsu.

- Nikdy
- Zriedka
- Občas
- Často
- Vždy

33. Cítim úzkosť a/alebo trpím záchvatmi paniky. *

Označte iba jednu elipsu.

- Nikdy
- Zriedka
- Občas
- Často
- Vždy

34. Rušia ma hluk a davy ľudí. *

Označte iba jednu elipsu.

- Nikdy
- Zriedka
- Občas
- Často
- Vždy

Psychosomatické ťažkosti

35. Trpím búšením srdca alebo bolesťou na hrudníku. *

Označte iba jednu elipsu.

- Nikdy
- Zriedka
- Občas
- Často
- Vždy

36. Trpím žalúdočnými a/alebo červnými ťažkosťami. *

Označte iba jednu elipsu.

- Nikdy
- Zriedka
- Občas
- Často
- Vždy

37. Trpím bolesťami hlavy. *

Označte iba jednu elipsu.

- Nikdy
- Zriedka
- Občas
- Často
- Vždy

38. Trpím bolesťou svalov, napríklad krku, ramena alebo chrbta. *

Označte iba jednu elipsu.

- Nikdy
- Zriedka
- Občas
- Často
- Vždy

39. Bývam chorý/á. *

Označte iba jednu elipsu.

- Nikdy
- Zriedka
- Občas
- Často
- Vždy

Autorský dotazník

Technostres a kognitívna záťaž

40. Pociťujem úzkosť alebo napätie pri zavádzaní nových verzií diagnostického softvéru (PACS/RIS) a novej prístrojovej techniky. *

Označte iba jednu elipsu.

- Nikdy
- Zriedka
- Občas
- Často
- Vždy

41. Množstvo artefaktov a technických chýb pri vyšetreniach vnímam ako frustrujúci faktor, ktorý spomaľuje moju prácu. *

Označte iba jednu elipsu.

- Nikdy
- Zriedka
- Občas
- Často
- Vždy

42. Mám pocit, že očakávania na moju rýchlosť práce rastú rýchlejšie než výkonnosť technológií, ktoré používam.*

Označte iba jednu elipsu.

- Nikdy
- Zriedka
- Občas
- Často
- Vždy

Fyzikálne faktory a ergonómia

43. Práca v prostredí s nedostatkom prirodzeného denného svetla negatívne ovplyvňuje moju náladu a energiu. *

Označte iba jednu elipsu.

- Nikdy
- Zriedka
- Občas
- Často
- Vždy

44. Počas pracovnej zmeny pociťujem výraznú vizuálnu únavu (pálenie očí, rozmazané videnie) v dôsledku sledovania monitorov. *

Označte iba jednu elipsu.

- Nikdy
- Zriedka
- Občas
- Často
- Vždy

45. Hluk prístrojov (najmä pri MR) u mňa vyvoláva pocit vnútornej nepohody. *

Označte iba jednu elipsu.

Nikdy

Zriedka

Občas

Často

Vždy

46. Neustála prítomnosť klimatizácie u mňa vyvoláva pocit vnútornej nepohody. *

Označte iba jednu elipsu.

Nikdy

Zriedka

Občas

Často

Vždy

47. Mám pocit, že vybavenie pracovísk (stoličky, stoly, monitory, ovládacie prvky počítačov) mi nevyhovuje. *

Označte iba jednu elipsu.

Áno

Nie

48. Pri práci vstupujem k aktívnemu zdroju žiarenia (napríklad skiaskopické vyšetrenia, skiagrafia na operačnom sále, intervenčná rádiológia) *

Označte iba jednu elipsu.

- Áno
- Nie

49. Mám pocit, že opatrenia na moju ochranu pred žiarením včítane osobných ochranných prostriedkov nie sú dostatočné. *

Označte iba jednu elipsu.

- Áno
- Nie

Pocit izolácie versus kontakt s pacientom

50. Chýba mi informácia o tom, ako dopadla liečba pacienta, na ktorého diagnostike/ liečbe som sa podieľal. *

Označte iba jednu elipsu.

- Nikdy
- Zriedka
- Občas
- Často
- Vždy

51. Cítim sa skôr ako 'servisný pracovník' pre iné klinické odbory než ako rovnocenný partner v liečebnom procese. *

Označte iba jednu elipsu.

- Nikdy
- Zriedka
- Občas
- Často
- Vždy

Stresory životného štýlu

52. Koľko 24 hodinových pohotovostných služieb slúžite priemerne mesačne? *

Označte iba jednu elipsu.

- 0
- 1 - 2
- 3 - 4
- 5 - 6
- 7 - 8
- 9 - 10
- 11 a viac

53. Koľko času v priemere za jeden týždeň trávite pri výpočtovej technike? *
(vrátane smartfónov a herných konzol)

Označte iba jednu elipsu.

- 1-10h
- 11-20h
- 21-30h
- 31-40h
- 41-50h
- 51-60h
- 70 a viac hodín

54. Prosím ak je to možné uveďte údaj "čas pred obrazovkou" z vášho smartfónu.

55. Mám pocit, že stres a nadmerné zaťaženie vedie k nasledovnému abúzu. (Je možné označiť viaceré možnosti alebo žiadnu, jedná sa o subjektívne hodnotenie)

Začiarknite všetky vyhovujúce možnosti.

- ☐ Prejedanie
- ☐ Nadmerné pitie kávy
- ☐ Fajčenie
